# Functional characterization of HIV-1 *Tat* exon-1 variants from North India and their implications on HIV-1 transactivation and TAR interaction

**DOI:** 10.1186/1471-2334-14-S3-P81

**Published:** 2014-05-27

**Authors:** Larance Ronsard, VG Ramachandran, Akhil C Banerjea

**Affiliations:** 1 Virology lab - II, National Institute of Immunology, New Delhi, India; 2 Department of Microbiology, UCMS and GTB Hospital, New Delhi, India

## Background

HIV-1 virus is a rapidly evolving virus due to genetic variability through its rapid replication, mutation and recombination potential which is a major hurdle in vaccine development. One of the effective ways to modulate HIV-1 infection is to target viral proteins; among the viral proteins, *Tat* plays a major role in HIV-1 pathogenesis. It activates viral gene expression through TAR interaction. The aim of this study was to characterize genetic and functional variants of *Tat* exon-1 from HIV-1 patients from North India.

## Methods

DNA was isolated from PBMCs and *Tat* exon-1 was PCR amplified with specific primers followed by cloning, sequencing and sequence analysis using bioinformatics tools for predicting subtypes, recombination events, conservation of domains and phosphorylation sites. Unique *Tat* exon-1 variants were functionally characterized for LTR transactivation, TAR interaction and cell death.

## Results

Genetic analysis of *Tat* exon-1 variants revealed 90% subtype C and 10% B/C recombinants, and the functional characterization showed varying levels of LTR transactivation, TAR interaction and cell death. A single mutation (S46F) in *Tat* exon-1 variants showed enhanced LTR transactivation through strong interaction with TAR.

## Conclusion

Possible role of *Tat* exon-1 variants in shaping the current HIV-1 epidemic in North India is discernible. Natural substitutions across the conserved functional domains were observed. There is evidence for the emergence of B/C recombinants within *Tat* exon-1. The impact of genetic variations in *Tat* exon-1 on its pivotal functions is apparent. These are likely to have implications for HIV-1 pathogenesis and strategies of vaccine formulations.

